# Robust Foot Clearance Estimation Based on the Integration of Foot-Mounted IMU Acceleration Data

**DOI:** 10.3390/s16010012

**Published:** 2015-12-23

**Authors:** Mourad Benoussaad, Benoît Sijobert, Katja Mombaur, Christine Azevedo Coste

**Affiliations:** 1 INRIA, LIRMM, Rue Saint Priest, Montpellier 34000, France; benoit.sijobert@inria.fr (B.S.); christine.azevedo@inria.fr (C.A.C.); 2 ORB, IWR, University of Heidelberg, Heidelberg 69117, Germany; katja.mombaur@iwr.uni-heidelberg.de

**Keywords:** foot clearance estimation, inertial measurement unit, 3D transformation, driftless integration

## Abstract

This paper introduces a method for the robust estimation of foot clearance during walking, using a single inertial measurement unit (IMU) placed on the subject’s foot. The proposed solution is based on double integration and drift cancellation of foot acceleration signals. The method is insensitive to misalignment of IMU axes with respect to foot axes. Details are provided regarding calibration and signal processing procedures. Experimental validation was performed on 10 healthy subjects under three walking conditions: normal, fast and with obstacles. Foot clearance estimation results were compared to measurements from an optical motion capture system. The mean error between them is significantly less than 15% under the various walking conditions.

## 1. Introduction

Foot clearance, defined as the foot elevation during the stride phase, is an important indicator of gait quality and safety in subjects with disabilities [1]. The minimum foot clearance is one of common gait deviations in people with stroke, which increase the risk of unsuccessful foot clearance (trips) and then of falls [2,3]. In a previous study, we showed that this parameter could be used to adapt the applied functional electrical stimulation in the context of drop foot correction in subjects with post-stroke hemiplegia [4]. In the present study, foot clearance (FC) is defined as the continuous heel height relative to the ground, whereas in other previous studies, maximum or minimum FC values during the stride phase were used instead [5].

Optical motion capture systems (OMCS) are the most commonly-used devices for measuring foot elevation during walking, providing high accuracy and direct measurement of the position of reflective markers located on body landmarks. However, these devices present multiple drawbacks, such as limited operating space and high cost, making their clinical use technically and financially difficult to implement.

The use of wearable devices, such as inertial measurement units (IMU) presents a promising alternative for gait analysis in outdoor and clinical contexts [6,7]. Such devices typically include three-axis accelerometers, three-axis gyroscopes and three-axis magnetometer sensors.

Raw data provided by IMU sensors requires complex post-processing, creating a source of errors in the estimation of IMU displacement and orientation. A major IMU raw data processing issue is the integration of acceleration signals and angular velocity signals (provided by gyroscopes). It is well known that the numerical integration of such data suffers from a high drift error rate, introducing significant errors in the estimation of IMU position and orientation. Magnetometers, on the other hand, provide an absolute orientation and do not require numerical integration. However, such systems can be disturbed by ferromagnetic materials, thus making this technique not reliable everywhere.

In the past few years, several teams have focused on the use and processing of IMU data for human motion analysis. Some authors have estimated IMU orientation based only on gyroscope data [8,9], or in combination with acceleration data [10], or acceleration and magnetometer data [10,11], using data fusion algorithms to overcome integration drift problems. To achieve driftless integration using gyroscope data alone, a weighted Fourier linear combiner (WFLC) algorithm [8] and an empirical mode decomposition (EMD) method [9] have been proposed for estimating the 3D orientation of the lower trunk during walking. Other authors have developed human biomechanical models to estimate joint angles [12] or walking parameters, such as stride length [13], based on IMU orientation estimates.

Only a few authors have focused on the estimation of IMU position or displacement based on acceleration data, mainly because such data require not single, but double integration, which makes the integration drift problem even more significant. In an effort to overcome this issue, an extended Kalman filter has been applied to fuse inertial (accelerometer and gyroscope) sensor data and magnetic sensor data for body position and orientation tracking [14].

Acceleration-based estimation of foot displacement during walking appears to be very complex due to the high accelerations involved. Therefore, only a few authors have investigated this approach. A simple biomechanical foot model (heel-toe distance) has been used to estimate maximum heel clearance and minimum toe clearance values with a mean error of approximately 4.1 cm and 1.3 cm, respectively [15]. These results were obtained with 12 healthy participants.

Only a few techniques not involving the use of a biomechanical foot model have been explored to avoid increased drift errors during walking [5,16,17,18,19,20]. Such a method has been applied to estimate maximum foot clearance values from double integration of acceleration data [16]. These authors used an optimally-filtered direct and reverse integration (OFDRI) technique based on frequency domain analysis of signals to be integrated [21]. Another published method is based on time domain analysis and the application of the zero velocity update (ZVU) principle. This method relies on the detection of foot-flat periods (*i.e.*, foot in contact with the ground) to set the velocity to a known constant value [17] or zero [18]. Such events can be detected using a foot-switch sensor [22] or based on the analysis of accelerometer and gyroscope data [23,24].

Based on the ZVU principle, integrated acceleration can be corrected using so-called “de-drifted” integration techniques [5,17]. Linear de-drifted integration has been applied in the case of five participants walking on a treadmill [17], whereas de-drifted integration based on a p-chip interpolation function has been used to estimate maximum foot clearance values with a mean error of approximately 1.9 cm [5]. ZVU-based integration correction based on extended Kalman filtering has also been proposed [17,18].

According to another proposed ZVU-based correction method [19,20], the sensor error was modeled as a constant acceleration bias in the sensor coordinate system and corrected backwards based on the estimated error observed at each detected event. In these two studies, ZVU-based correction was applied to estimate driftless velocities and thereby calculate the walking speed over the stride. However, displacements were not estimated.

All of these cited works do not go further to get the foot clearance, directly integrated the velocity without any correction or estimated only the maximal foot clearance instead of a curve of foot clearance, which is required in our work. In the present study, we focused on the double integration of foot acceleration data from a foot-mounted IMU. We therefore used the ZVU principle to limit the increase in integration drift between two successive strides and then applied the above-mentioned acceleration correction method [19,20] to each stride. For displacement estimation purposes (particularly foot clearance estimation), we adapted the same principle to vertical velocity integration and correction, by generalizing to a zero displacement update (ZDU) principle, where walking is assumed as occurring on flat and horizontal ground. In accordance with our previous work and with clinical context requirements [4], our goal was to obtain a foot clearance estimation error of less than 2 cm (minimum foot clearance [3]) and to make our method robust to misalignment of IMU axes and foot axes. The proposed IMU-based foot clearance estimation algorithm is detailed in Section 2 below. Experimental setup and validation methods are described in Section 3. Estimation results and the performance evaluation are presented and discussed in Section 4. Conclusions and future prospects are given in Section 5.

## 2. IMU-Based Foot Clearance Estimation

**Figure 1:**
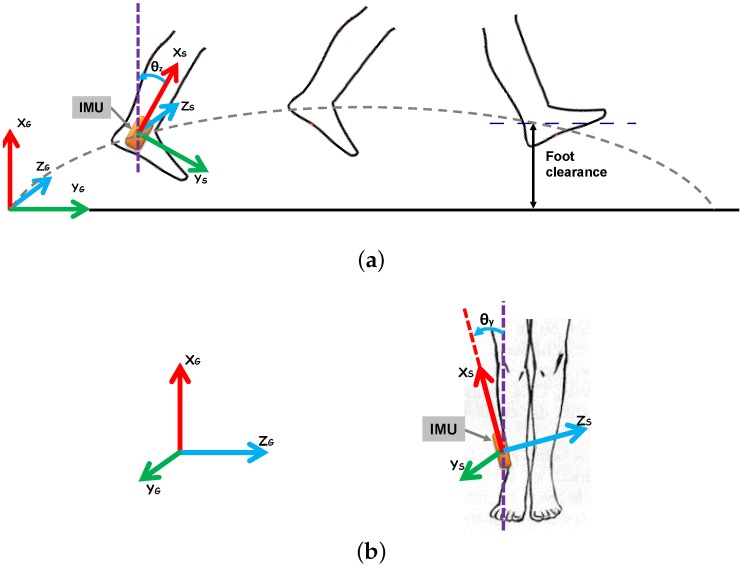
IMU placement and frame transformation in (**a**) the sagittal view and (**b**) the frontal view.

Figure 1 shows sagittal and frontal views of IMU placement for foot clearance estimation (FCE). Possible misalignment of the IMU with respect to the vertical axis is also illustrated (Figure 1b). As mentioned earlier, we defined foot clearance as the continuous heel clearance (Figure 1a). Estimation of ankle joint clearance (where the IMU is placed) is adequate, since there is only one bias to be added, corresponding to the distance between IMU-based estimated clearance and heel clearance. Moreover, this ankle joint clearance is most appropriate in the context of drop foot correction [4].

**Figure 2:**
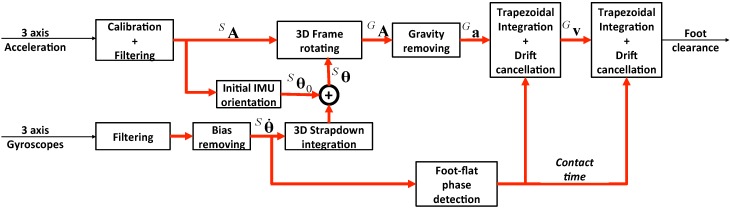
IMU-based foot clearance estimation algorithm.

The proposed FCE algorithm (illustrated in Figure 2) uses only acceleration in the global frame. However, since acceleration is measured in the sensor frame, gyroscope-based angular rate data are used to estimate foot acceleration in the global frame. Each step of the method is detailed below.

### 2.1. Accelerometer Calibration

An efficient calibration procedure is essential for ensuring accurate accelerometer-based acceleration measurement. The proposed calibration procedure is based on a previous study [25] and consists of optimizing the three-axis accelerometer output, such that, under stationary conditions, the norm of the acceleration vector is equal to the norm of the gravity vector (*i.e*., 9.81 m/s${}^{2}$). For this purpose, the IMU sensor was placed in N (>20) different random orientations, and each corresponding acceleration vector was measured under static conditions. The mathematical model of calibrated accelerometer output is described as follows [25]:

(1) $$A=S(A_{m}-B)$$

where A is the calibrated acceleration, $A_{m}$ is the measured acceleration prior to calibration and S and B are, respectively, the scale matrix and bias vector identified during the calibration procedure. S and B were identified using a Newton iterative optimization algorithm that minimizes quadratic errors between the norm of the calibrated acceleration vector and the norm of the gravity vector for each static orientation. This calibration was performed once, and the resulting calibration matrices (S, B) were used to correct measured acceleration data (Figure 2) using Equation (1).

### 2.2. Data Filtering and Bias Removing

Since IMU data were post-processed offline, there were no real-time constraints. We therefore chose a data acquisition frequency of 200 Hz. However, frequency sampling should be optimized for future real-time applications, so as to ensure sufficient measurement quality and compliance with real-time constraints.

Three-axis gyroscope data were band-pass filtered between 0.001 Hz and 5 Hz using a first-order Butterworth filter to remove constant and high frequency components. The angular rate in the stationary state (subjects were asked to remain static at the start of each trial) was considered as a bias and, therefore, automatically detected, quantified and removed from the entire gyroscope signal. For three-axis acceleration data, a first-order low-pass Butterworth filter with a 10-Hz cutoff frequency was applied to remove high-frequency noise. However, the bias was not subtracted from the acceleration signal at this stage, since it corresponds to the gravity acceleration, which changes according to IMU orientation.

### 2.3. Gyroscopic Data Integration

As illustrated in Figure 2, processing of gyroscopic data is crucial for processing of gravity-free acceleration (due to the movement only), since the measured acceleration also includes the gravity effect. Therefore, proper processing and integration of gyroscopic data was required to expect proper acceleration-based displacement estimation (Section 2.4).

The filtered angular rate ${}^{S}\dot{\theta }$ (see Figure 2) was then integrated using a 3D strapdown integration method [11,26]. This integration method has the advantage of considering rotations around all three axes, since IMU rotation exclusively in the sagittal plane cannot be ensured during walking. This is based on a recursive equation (Equation (2)) that updates the 3D orientation matrix of the IMU:

(2) $${}^{G}R_{i}{=}^{G}R_{i-1}{+}^{G}R_{i-1}[\Delta t\cdot {{}^{S}\dot{\theta }}_{i}\times ]$$

where ${}^{G}R_{i}$ is the 3D orientation matrix in the global frame at sample *i*, the initial transformation matrix ${}^{G}R_{0}$ is the identity matrix (*I*), Δt is the time interval between two successive samples (i-1, *i*) and ${{}^{S}\dot{\theta }}_{i}$ is the angular rate vector at sample *i*. [a×] is the skew-symmetric matrix representing the cross-product operator, such that:

(3) $$\left[a\times \right]=\left[\begin{array}{ccc} 0 & -a_{z} & a_{y}\\ a_{z} & 0 & -a_{x}\\ -a_{y} & a_{x} & 0 \end{array}\right]$$

where $a_{x}$, $a_{y}$, $a_{z}$ are the elements of a.

At each time instant, the transformation matrix ${}^{G}R_{i}$ represents the IMU frame transformation with respect to the initial IMU configuration. In practice, IMU orientation is never absolutely vertical in the initial position, and as a result, the initial frame is not the global frame. Therefore, in order to define the transformation matrix with respect to a global frame independently of initial placement, we made the initial matrix ${}^{G}R_{0}$ equal to a transformation matrix between the initial IMU position and the global frame (vertical orientation).

This initial transformation matrix consists of a rotation about the Z-axis (pitch) with an angle of $\theta_{z0}$, followed by another rotation about the Y-axis (roll) with an angle of $\theta_{y0}$, where the sign of rotation is as shown in Figure 1. This initial transformation matrix is therefore defined as follows:

(4) $${}^{G}R_{0}=\left[\begin{array}{ccc} cos(\theta_{z0}) & sin(\theta_{z0}) & 0\\ -sin(\theta_{z0}) & cos(\theta_{z0}) & 0\\ 0 & 0 & 1 \end{array}\right]\cdot \left[\begin{array}{ccc} cos(\theta_{y0}) & 0 & sin(\theta_{y0})\\ 0 & 1 & 0\\ -sin(\theta_{y0}) & 0 & cos(\theta_{y0}) \end{array}\right]$$

where $\theta_{z0}$ and $\theta_{y0}$ were estimated based on the projection of the gravity vector on the three axes [11], measured by accelerometers during the stationary period, *i.e*., prior to the start of movement. Considering the order of rotation defined above (Equation (4)), these initial angles (tilt angles) were calculated using the following equations:

(5) $$\theta_{z0}=\arctan\left(\frac{{}^{S}G_{Y}}{{}^{S}G_{X}}\right)\;\;\theta_{y0}=\arctan\left(\frac{{}^{S}G_{Z}}{\sqrt{{}^{S}G_{X}^{2}{+}^{S}G_{Y}^{2}}}\right)$$

where ${}^{S}G=\left(\begin{array}{ccc} {}^{S}G_{X} & {}^{S}G_{Y} & {}^{S}G_{Z} \end{array}\right)$ is the gravity vector measured on the three IMU axes during the stationary period. Based on our measurements, the drift in gyroscopic data integration was neglected, and as a result, no drift cancellation was required at this level.

### 2.4. Displacement Estimation

The proposed displacement estimation method is based on double integration of foot acceleration data on the global frame axes. To achieve this objective and thereby overcome the integration drift problem, the following steps are applied.

#### 2.4.1. 3D Frame Transformation and Gravity Removing

It is well known that IMU-based acceleration measurements correspond to the acceleration in the sensor frame, whereas the useful acceleration should be the acceleration with respect the global frame. Moreover, the measured acceleration includes gravitational acceleration, which should be removed to obtain gravity-free acceleration due to movement only. Therefore, the acceleration in the sensor frame ${}^{S}A$ was first transformed to the global frame (${}^{G}A$ in Figure 2) using the strapdown transformation matrix (Equation (2)):

(6) $${}^{G}A{=}^{G}R_{i}{*}^{S}A$$

Gravitational acceleration was then removed to obtain the gravity-free acceleration in the global frame ${}^{G}a$:

(7) $${}^{G}a{=}^{G}A-\left(\begin{array}{c} 9.81\\ 0\\ 0 \end{array}\right)$$

To obtain the vertical foot displacement (foot clearance), the vertical component of the gravity-free acceleration ${}^{G}a$ needs to be integrated twice (Equation (7)).

#### 2.4.2. Foot-Flat Phase Detection

One of the main problems with the integration of acceleration data is the significant integration drift, which is further increased by double integration. The proposed solution to overcome this problem is an adaptation of the ZVU principle [27]. The proposed ZDU principle consists of detecting when the foot is flat on the ground and setting the vertical velocity and displacement to zero. These particular events are detected by the foot-flat phase detection algorithm (illustrated in Figure 2), which uses the angular rate about the Z-axis (${}^{S}{\dot{\theta }}_{z}$) to automatically detect when the foot is flat on the ground [20,28]. Such events are detected based on the detection of the minimum angular velocity at each stride cycle. This foot-flat phase corresponds to the period of time between heel-strike and heel-off.

#### 2.4.3. Drift Cancellation

The foot-flat phase detection algorithm segments walking into several strides. This segmentation avoids the accumulation of drift error between different strides. However, at the end of each stride, a local drift (occurring only during a current stride) appears as an error between integrated and theoretical data, corresponding to the foot-flat phase. In the proposed method, we applied a drift cancellation on the vertical foot velocity for each stride, based on the error accumulated at the end of each stride and the zero vertical velocity assumption. This correction used the following model [19]:

(8) $$V_{x\_corrected}\left(t\right)=V_{x}\left(t\right)-\frac{V_{x}\left(T\right)}{T}t$$

where $V_{x}\left(t\right)$ is the vertical foot velocity obtained using trapezoidal integration of gravity-free acceleration on the X-axis (${}^{G}a_{x}$), $V_{x}\left(T\right)$ is the calculated velocity at the end of the stride phase, *t* is the time instant of the current stride, *T* is the entire duration of the current stride and $V_{x\_corrected}\left(t\right)$ is the vertical foot velocity after correction for the entire stride phase.

To estimate the vertical foot displacement (foot clearance), we first integrated the driftless corrected vertical velocity ($V_{x\_corrected}$) and then applied the same drift cancellation on this vertical foot displacement, assuming zero foot clearance at the end of the stride (foot-flat phase). This second correction used the following model:

(9) $$D_{x\_corrected}\left(t\right)=D_{x}\left(t\right)-\frac{D_{x}\left(T\right)}{T}t$$

where $D_{x}\left(t\right)$ is the vertical foot displacement obtained by trapezoidal integration of the corrected velocity $V_{x\_corrected}\left(t\right)$, $D_{x}\left(T\right)$ is the calculated displacement at the end of the stride phase and $D_{x\_corrected}\left(t\right)$ is the vertical foot displacement after correction during the stride phase, corresponding to the estimated foot clearance.

Since the drift cancellation was applied during the swing phase, the foot clearance was set to zero during the foot-flat phase in agreement with our assumptions.

## 3. Experimental Validation

For validation of our foot clearance estimation method, an experimental procedure was applied on both feet of ten healthy participants whose characteristics are summarized in Table 1 below.

**Table 1:** Characteristics of study participants.

Subject Number	Age (Years)	Height (m)	Sex (M/F)
1	35	1.85	M
2	24	1.89	M
3	40	1.60	F
4	23	1.55	F
5	23	1.71	F
6	29	1.93	M
7	26	1.72	M
8	26	1.62	F
9	24	1.76	M
10	23	1.58	F

**Figure 3:**
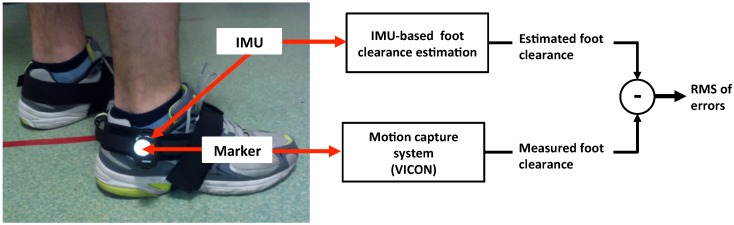
Experimental setup and validation of IMU-based foot clearance estimation.

Two IMU sensors (HikoB Fox©Villeurbanne, France) were strapped to both feet of each subject (at the ankle joint), as shown in Figure 3.

Each subject stood upright, remained still for a few seconds and then walked a distance of approximately 10 m on flat horizontal ground. Each subject covered this distance four times walking normally, four times walking fast and two times crossing obstacles. The fast walk was to test a higher level of acceleration and then to increase the drift level. The purpose of walking with obstacles was to test the estimation of a higher level of clearance and nonsymmetrical and nonperiodic walking. The obstacle heights range from 20 cm to 50 cm and were placed to influence normal walking, increase the foot clearance and test the robustness of our estimation method. As illustrated in Figure 3, foot clearance estimation was validated with a BONITA VICON motion capture system, using reflective markers located on the IMUs.

Measured and estimated peak foot clearance values were automatically detected and used to synchronize the two datasets. Foot clearance data for the entire movement, measured with the motion capture system and estimated using the proposed method, was then compared (Figure 3). Subsequently, the RMS value of foot clearance errors (RMSE) was quantified for the evaluation of foot clearance estimation performance, using the following criterion:

(10) $$RMSE=\sqrt{\frac{1}{N}\sum_{i=1}^{N}{\left(X_{m}-X_{e}\right)}^{2}}$$

where $X_{m}$ and $X_{e}$ are respectively the measured and estimated foot clearance, discretized to *N*samples throughout the walking cycle duration. This number of samples (*N*) was associated with the walking cycle duration and discretization (sampling) frequency, *i.e*., approximately 200 Hz for both VICON and IMU signals.

To compare the foot clearance estimation performance under different walking conditions, it was necessary to calculate another error criterion, since foot clearance values obtained during walking with obstacles were three-times higher than during normal walking. A normalized RMSE percentage value was therefore calculated for each subject and walking type by normalizing the RMSE with respect to the maximum foot clearance during the current walking test as follows:

(11) $$NRMSE=\frac{RMSE}{max(X_{m})}\times 100$$

## 4. Presentation and Discussion of Results

### 4.1. Results

**Figure 4:**
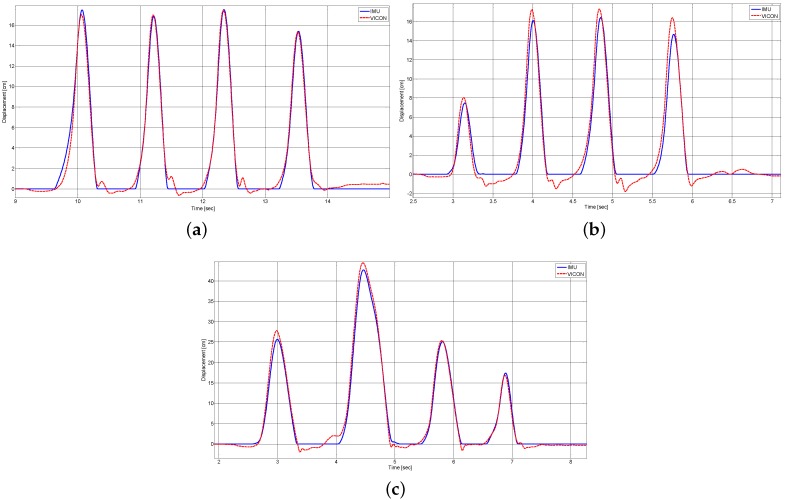
Foot clearance values measured by the VICON system and estimated by the IMU during: (**a**) Normal walking (Subject 1, left foot); (**b**) Fast walking (Subject 1, right foot); and (**c**) Walking with obstacles (Subject 10, right foot).

Figure 4 shows a typical set of foot clearance measurements obtained with a VICON motion capture system and compared to estimated values generated by the proposed algorithm (Section 2). Normal and fast walking data (Figure 4a,b) was obtained on the left and right foot, respectively, of Subject 1 (35 years old, 1.85 m), and walking-with-obstacles data (Figure 4c) were obtained on the right foot of Subject 10 (23 years old, 1.58 m).

**Figure 5:**
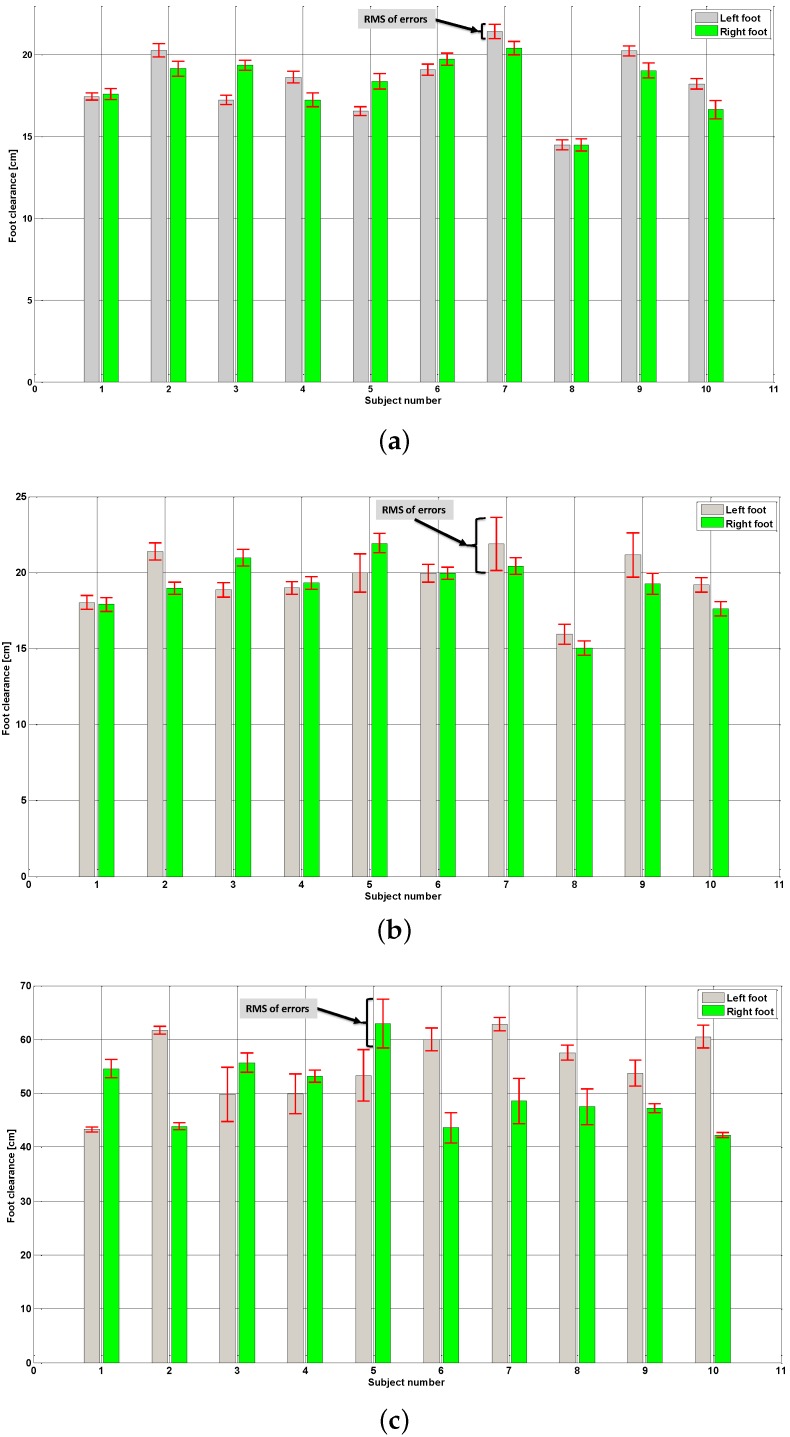
Maximum foot clearance and RMSE between VICON measurements and IMU-based estimates for both feet of each subject during: (**a**) Normal walking; (**b**) Fast walking; and (**c**) Walking with obstacles.

Foot clearance measurements obtained with the VICON system and estimated using the IMU-based algorithm for both feet of all 10 participating subjects are shown as histograms in Figure 5.

These three histograms correspond to results obtained during normal walking (Figure 5a), fast walking (Figure 5b) and walking with obstacles (Figure 5c). In each histogram, the height of each bar corresponds to the maximum foot clearance measured with the VICON system on each foot of each subject. The bracket centered at the top of each bar represents the RMS value of foot clearance errors (Equation (10)) between VICON measurements and the proposed algorithm.

The individual step error rate between VICON measurements and IMU-based foot height estimates was analyzed using a Student *t*-test. Benjamini–Hochberg correction was applied to limit the false positive rate. The mean error is significantly (*p*-value < 0.05) lower than 15% under the various walking conditions. A Spearman test showed that walking speed affects the error-rate.

The results shown in Figure 4 exhibit very good correspondence between foot clearance values estimated with the proposed method and those measured with the VICON motion capture system. In these particular examples, the good results obtained are illustrated by a low RMS of foot clearance estimation errors, *i.e*., approximately 0.35 cm during normal walking (Figure 4a), approximately 0.62 cm during fast walking (Figure 4b) and approximately 0.89 cm during walking with obstacles (Figure 4c).

Results obtained in all subjects and summarized in Figure 5 show on the whole a good estimation of foot clearance compared to a measured one with VICON motion capture, which is considered as a reference due to its high accuracy. Indeed, the average of all normalized errors (NRMSE) is approximately 6.5%, and in 96% of all experiment cases, these normalized errors are less than 15%. In addition, in 83% of all experiment cases, these normalized errors are less than 10%, which correspond to 2 cm if the maximum foot clearance is approximately 20 cm.

The results obtained during the normal walking experiments, summarized by the histogram in Figure 5a, show that all RMS values of foot clearance estimation errors are less than 1.13 cm, whereas the average value of all of these errors is approximately 0.74 cm. This shows very good foot clearance estimation using the proposed method during normal walking for various subjects with different heights.

The estimation results declined during the fast walking experiments (Figure 5b), since the average of RMS errors increase to 1.3 cm, whereas there maximum value is approximately 3.5 cm (left foot, Subject 7). However, even in these experiment sets, the results are good, since in 85% of the experiments, the RMS errors remained less than 1.5 cm with an average of RMS errors around 1 cm.

Walking with obstacles is experimented on here to test the limits and the strength of our foot clearance estimation method. The results of these experiments for all subjects, summarized in Figure 5c, show larger RMS values of foot clearance estimation, which reached 10 cm (right foot, Subject 3). However, these results in cm cannot be compared to the previous results, since the maximum foot clearances are not comparable. Therefore, normalized RMS errors (Equation (11)) are used hereinafter for the purposes of comparison and discussion.

Table 2 summarizes the subject average speed during each walking type using VICON data measurements. This brings numerical information about speed and, thereby, acceleration levels involved during each walking type.

**Table 2:** Subject average speed during each walking type.

Subject Number	Walking Speed (m/s)		
	Normal	Fast	With Obstacles
1	1.05	1.71	1.07
2	1.21	1.83	1.09
3	0.95	1.66	0.70
4	1.21	1.61	1.06
5	0.97	1.84	1.21
6	0.94	1.33	1.02
7	1.05	1.50	1.04
8	0.93	1.71	0.85
9	1.00	1.81	1.18
10	1.01	1.42	0.98

### 4.2. Discussion

From the results, in walking with obstacles, 85% of the normalized RMS errors are lower than 15%. The average value of NRMSE in these 85% is approximately 6.9%, which correspond to 1.4 cm if the maximum foot clearance is approximately 20 cm. This result is therefore comparable to previous results and shows better foot clearance estimation performance if we compare it to the average RMS errors obtained in the fast walking case (1.3 cm). The estimation performance decrease in this case cannot be related to the speed difference with normal walking, since their speeds are close (see Table 2). This performance degradation is mainly due to the foot-flat phase detection algorithm (Figure 2), which presents some limits when the walking gait is not very natural, as in the case of walking with obstacles. Indeed, the accuracy of walking segmentation has an important impact on the foot clearance estimation, which decreased the estimation performances in the case of walking with obstacles.

In the foot-flat phase detection algorithm, the detection of the first events can be a source of errors in the case of the foot, which stands at the beginning, when the other foot begins the swing phase. Indeed, the motion start of this foot, detected from gyroscope data, is not the real start, since the ankle rotates around the heel before heel-off. To overcome this problem, we have combined the segmentation method with the linear acceleration start to make the detection of this first event more robust. Assuming that each subject starts walking with the same foot for all experiments, it seems interesting to compare for each subject the difference between the RMS errors obtained on the right and left foot. The average of all subjects of absolute variation between right and left foot estimation error is approximately 0.19 cm during normal walking and approximately 0.63 cm during fast walking. This small variation shows the robustness of the proposed segmentation to the choice of starting foot during walking. This comparison cannot be made in the case of walking with obstacles, since obstacles make the gait nonsymmetrical, and thus, the error produced in the right foot and left foot cannot be compared.

In addition to its good accuracy, the originality of the proposed foot clearance estimation method lies in its robustness to errors in IMU sensor placement on the foot around the Y-axis and Z-axis (Figure 1). This was facilitated by initial tilt angles (Equation (5)) measured under initial stationary conditions and included in the gravity-free acceleration calculation.

As mentioned in the experimental validation section (Section 3), data obtained from the IMU sensors cannot be used online. This IMU sensors technology was however enough to validate our method and quantify its performance by comparing results to measurements with the VICON motion capture system, which is known for its high accuracy.

As in the current work, we assumed walking occurs on a flat and horizontal floor, some modifications should be made in the future in order to consider other types of walking, like climbing stairs. In this case, the height of each stair should be known and included in the correction model (Equation (9)) Another future prospect consists of the online estimation of the foot clearance. This requires using another IMU technology.

The difference in accuracy estimation between normal and fast walking can be due to the limit of the acquisition frequency in the presence of a higher acceleration, related to a higher walking speed (see Table 2). A study comparing the acquisition frequency and its effect on different walking speeds can be interesting to explore and is planned in future works.

## 5. Conclusions

In the present work, a single mounted inertial measurement unit (IMU) was used to estimate the foot clearance, from the double integration and de-drift correction of acceleration. An original algorithm of foot clearance estimation is proposed, which is robust to misalignment of IMU axes with respect to the foot axes. Our foot clearance estimation method was validated on both feet of 10 healthy subjects, with different sizes and gaits (normal walking, fast walking and walking with obstacles). For this experimental validation, an optical measurement of IMU position through a motion capture system was used as the reference. Then, the deviation between the IMU-based foot clearance estimation and the one measured with the motion capture system was quantified and discussed.

Experimental validation results obtained for all subjects showed good foot clearance estimation performance, where the average of all normalized RMSE values, including all walking types, is approximately 6.5%, which corresponds to 1.3 cm, if the maximum foot clearance is approximately 20 cm.

Detailed results showed the best estimation performance during normal walking, with an average of RMS error around 0.74 cm, where this average of RMS errors is around 1.3 cm in the case of the fast walking. This performance degradation can be due to the limitation of acquisition frequency in the presence of a higher acceleration. The results obtained during walking with obstacles show a decrease in estimation performance, even when normalized with a higher foot clearance than for other walking types. However, even in this case, the average of normalized RMS errors in 85% of experiments is approximately 6.9%, which presents a good estimation performance. This performance loss can be due to a decrease of segmentation walking accuracy, since this walking type is not as usual and periodic as normal and fast walking types.

Our foot clearance estimation method was performed offline, since IMU data were acquired locally on the SD card of the sensor. Regarding potential prospects for IMU wireless data transmission, an adaptation of this method will be required for an online version of the proposed foot clearance estimation algorithm.

Some of the proposed algorithms are included in a Python toolkit devoted to developments regarding acquisition, analysis and visualization of IMU-based motion capture. Open access to this toolkit is available via the link [29].
